# L-glutamine decreases the severity of mucositis induced by chemoradiotherapy in patients with locally advanced head and neck cancer: A double-blind, randomized, placebo-controlled trial

**DOI:** 10.3892/or.2014.3564

**Published:** 2014-10-23

**Authors:** TAKAE TSUJIMOTO, YOSHIFUMI YAMAMOTO, MASAFUMI WASA, YUKINORI TAKENAKA, SUSUMU NAKAHARA, TASTUYA TAKAGI, MAMIKO TSUGANE, NORIYUKI HAYASHI, KAZUHISA MAEDA, HIDENORI INOHARA, ETSUKO UEJIMA, TOSHINORI ITO

**Affiliations:** 1 Department of Complementary and Alternative Medicine, Osaka University Graduate School of Medicine, Suita, Osaka, Japan; 2 Department of Clinical Pharmacy Research and Education, Osaka University Graduate School of Pharmaceutical Sciences, Suita, Osaka, Japan; 3 Department of Clinical Pharmacy, Faculty of Pharmaceutical Sciences, Kobe Gakuin University, Kobe, Hyogo, Japan; 4 Department of Otorhinolaryngology-Head and Neck Surgery, Osaka University Graduate School of Medicine, Suita, Osaka, Japan; 5 Department of Pediatric Surgery, Osaka University Graduate School of Medicine, Suita, Osaka, Japan; 6 Department of Pharmainformatics and Pharmacometrics, Osaka University Graduate School of Pharmaceutical Sciences, Suita, Osaka, Japan

**Keywords:** glutamine, mucositis, chemoradiotherapy, head and neck cancer, clinical trial, supportive care, adverse effect, nutrition, quality of life, pharynx, larynx

## Abstract

The incidence of severe mucositis in the oral cavity, pharynx and larynx is high among patients with head and neck cancer (HNC) receiving chemoradiotherapy (CRT), resulting in significant pain and impairment of quality of life. The present study investigated whether L-glutamine (glutamine) decreases the severity of mucositis in the oral cavity, pharynx and larynx induced by CRT. This double-blind, randomized, placebo-controlled trial included 40 untreated patients with squamous cell carcinoma of the nasopharynx, oropharynx, hypopharynx or larynx. Patients received 66 or 70 Gy of total radiation at the rate of 2 Gy/fraction daily and 5 fractions/week. Cisplatin (20 mg/m^2^) and docetaxel (10 mg/m^2^) were intravenously co-administered once a week for 6 weeks. Patients were randomized to orally receive either glutamine (group G) or placebo (group P) at a dose of 10 g 3 times a day throughout the CRT course. Mucositis was assessed using the National Cancer Institute Common Terminology Criteria for Adverse Events version 3.0. The primary end point was mucositis severity. Mucositis developed in all patients. A maximal mucositis grade of G4 was observed in 0 and 25% group G and P patients, respectively, while that of G2 was observed in 10 and 0% group G and P patients, respectively (p=0.023). Glutamine significantly decreased the maximal mucositis grade (group G, 2.9±0.3; group P, 3.3±0.4; p=0.005) and pain score at weeks 4, 5 and 6. Glutamine significantly decreased mucositis severity in the oral cavity, pharynx and larynx induced by CRT in patients with HNC.

## Introduction

Chemoradiotherapy (CRT) has led to the local control of tumor growth and an improved survival rate in patients with head and neck cancer (HNC) (1). However, a high incidence of severe mucositis over a wide area of the oral cavity, pharynx and larynx is usually induced by CRT. Patients frequently become unable to consume oral medications due to severe mucositis. Therefore, physicians are sometimes compelled to decrease the chemotherapy dose or discontinue radiation, which can adversely affect treatment outcomes (2). To date, several efforts have been made for the prevention and treatment of severe mucositis, such as oral care, topical anesthetic use, antimicrobial agent use and oral rinsing; however, no standard therapy has been established (3–5).

Glutamine is the most abundant amino acid in our body. It is a primary fuel and an essential precursor of nucleotide biosynthesis in rapidly proliferating cells such as enterocytes, fibroblasts, lymphocytes and macrophages (6–9). Therefore, glutamine is classified as a conditionally essential amino acid in these cell types (10). Furthermore, glutamine serves as a glutathione synthesis substrate and exhibits antioxidant properties. When exposed to severe stress, the body fails to synthesize sufficient amounts of this amino acid, resulting in decreased plasma glutamine levels. Under these conditions, mucosal immunity is negatively affected and glutamine release from muscle tissues is decreased. Patients with advanced cancer undergoing cytotoxic therapy reportedly develop glutamine deficiency (11).

Over the last 2 decades, several studies have tested whether glutamine decreases the incidence and severity of chemotherapy-, radiotherapy- or CRT-induced mucositis (12–17). However, the study design of some clinical trials was not adequate to evaluate the role of glutamine in the prevention or treatment of mucositis, thereby leading to conflicting results (14–16). The quality of these studies was insufficient since they included a small patient sample, were poorly designed and used an inadequate glutamine dose; therefore, they were unable to evaluate the effectiveness of glutamine against mucositis. In addition, all studies conducted previously observed only oral mucositis (OM). Patients with HNC receiving CRT developed mucositis not only in the oral cavity, but also in the pharynx and larynx. A double-blind, randomized, placebo-controlled trial was required to clarify this. The present study assessed whether glutamine decreases the severity of CRT-induced mucositis in patients with HNC.

## Materials and methods

### Patients

Participants with pathologically diagnosed, primary squamous cell carcinoma of the nasopharynx, oropharynx, hypopharynx or larynx who were scheduled to undergo CRT were included. Forty patients between 38 and 77 years of age (median, 63.5 years) were included in the present study. Of these, 85% (34/40) were males and 15% (6/40) were females. Patients with active mouth or throat soreness before treatment, uncontrolled diabetes mellitus, or severe renal or hepatic insufficiency were excluded.

### Study design

This double-blind, randomized, placebo-controlled trial was conducted in accordance with the Helsinki Declaration. The protocol and informed consent form were reviewed and approved by the Institutional Review Board of Osaka University Hospital (ID no. 09180). All patients provided written informed consent before undergoing any study-related procedures. The present study was registered with the University Hospital Medical Information Network Clinical Trials Registry (UMIN000003991).

The patients were consecutively enrolled from May 19, 2010 to June 17, 2013. An independent observer not involved in the study conduct randomly allocated eligible patients to either the glutamine group (group G) or the placebo group (group P). The assignment of patients was randomized by gender, age (<65 or ≥65 years) and tumor location (nasopharynx/oropharynx or hypopharynx/larynx). Glutamine, which is a dietary supplement (Emmaus Medical, Inc., Torrance, CA, USA), and placebo (Matsutani Chemical Industry Co., Ltd., Suita, Osaka, Japan) were received by 20 patients each. The patients also received 66 or 70 Gy of total radiation at 2 Gy/fraction daily and 5 fractions/week. Cisplatin (20 mg/m^2^) and docetaxel (10 mg/m^2^) were intravenously co-administered once a week for 6 weeks. The patients orally consumed 10 g of either glutamine or placebo 3 times a day (at 7:00, 11:00 and 16:00 h) throughout the CRT course. When patients were unable to consume glutamine or placebo orally due to severe mucositis, we dissolved the agent in water and administered it via a feeding tube 30 min before tube feeding was started. All patients and medical staff, including physicians, nurses, pharmacists, nutrition support team (NST) members and investigators, were in compliance with the double-blind design.

The primary end point was the severity of mucositis [National Cancer Institute Common Terminology Criteria for Adverse Events version 3.0; (NCI CTCAE ver. 3.0)]. The secondary endpoints were duration and time to mucositis onset, patient-reported pain score according to a numerical rating scale (NRS), incidence and duration of opioid use, total opioid dose, incidence and duration of nutritional supplementation, and clinical data. The safety end points were the adverse effect incidence, abnormal laboratory findings and amino acid imbalance.

### Clinical and nutritional support

All patients had completed dental and oral examination before treatment and were made to undergo oral care under a nurse’s supervision throughout CRT. The NST assessed all patients weekly and managed their nutritional status. When patients could not manage an adequate oral diet due to severe mucositis, nutrition supplementation was provided through feeding tubes.

### Data collection

Mucositis was evaluated by images of laryngoscope using NCI CTCAE ver. 3.0. Two well-trained physicians from the Department of Otorhinolaryngology assessed mucositis in the oral cavity, pharynx and larynx once a week with a naked eye and laryngoscope until patient discharge. When grades of mucositis varied from site to site, the highest grade was chosen for the analysis. Hematological and blood chemistry tests were performed at baseline, once a week during the study treatment (weeks 1–6), and after treatment (weeks 7–9) or until patient discharge, whichever occurred first. The NRS was used as a patient-reported pain scale. Patients assessed the strongest pain experienced during a day on a 0–10 scale. The primary treatment outcome was evaluated 10 weeks after the completion of treatment using computed tomography (CT), positron emission tomography/CT (PET-CT) and biopsy.

### Statistical analysis

The sample size was obtained based on the following equation:

$$\text{N}≒2\cdot {\text{SD}}^{2}\cdot {\left({\text{Z}}_{\alpha }+{\text{Z}}_{\beta }\right)}^{2}/\Delta^{2}$$

N, sample size in each group; SD, standard deviation in each group; Δ, minimal difference between the populations; (Z_α_+Z_β_)^2^, index of power of test.

We found the sample size of 20 was required according to above equation. All data are expressed as means ± standard deviations.

NCI CTCAE grade and NRS scores were analyzed using the Mann-Whitney U test, and other continuous variables were analyzed using Welch’s t-test. Categorical variables were analyzed using Fisher’s exact test. The Kaplan-Meier method was used to analyze the time to onset of severe mucositis (≥G3) and the curves were compared using stratified log-rank tests. The p-values were two-sided and a value of <0.05 was considered to indicate a statistically significant difference. All statistical analyses were performed using JMP 11 software (SAS Institute, Inc.).

## Results

### Patient characteristics

**Figure 1:**
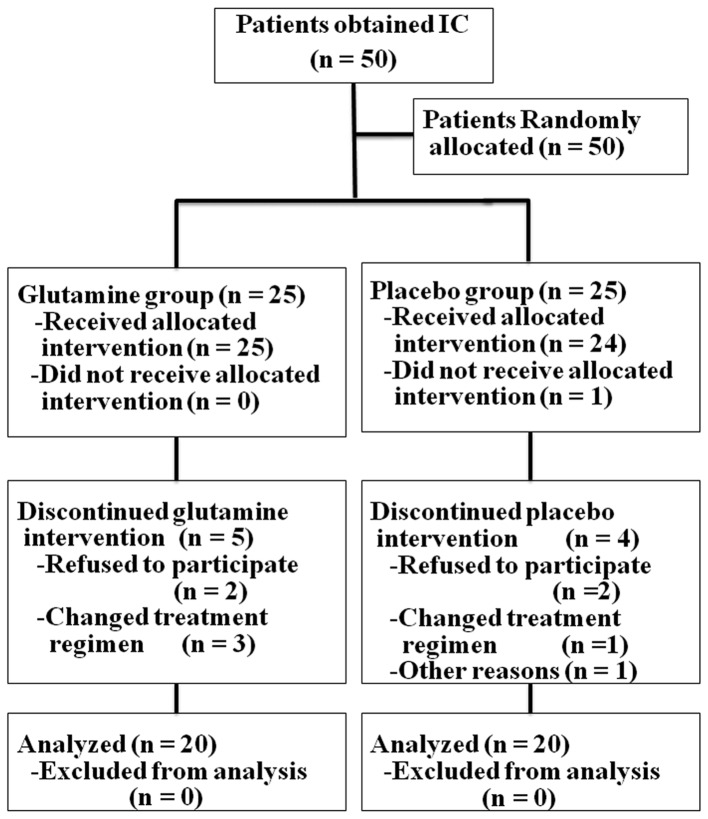
Consort diagram. IC, informed consent.

Fifty patients were enrolled between May 2010 and June 2013, with the last follow-up in August of 2013. Ten patients were excluded from analysis: 4 (group G, 3; group P, 1) whose treatment regimen was changed due to respiratory dysfunction or renal impairment during the CRT course, 4 (group G, 2; group P, 2) who refused to participate in the middle of the present study, and 2 (both from group P) who had to change from enteral to intravenous access. Hence, 40 patients were included in the complete analysis (Fig. 1). Baseline patient characteristics were similar between groups. There were 17 males and 3 females in each group (Table I). The average patient age was 60.5±10.8 years in group G and 63.2±5.4 years in group P. Patients in both groups were matched for primary tumor location, tumor stage, body mass index and Eastern Cooperative Group performance status. The mean total cisplatin dose was 193.3 and 187.2 mg and the mean total docetaxel dose was 97.7 and 93.9 mg in groups G and P, respectively. The mean total radiation dose was 66.4 and 66.2 Gy in groups G and P, respectively. The oral mucosa at baseline was healthy in all patients.

### Incidence and severity of mucositis

**Figure 2:**
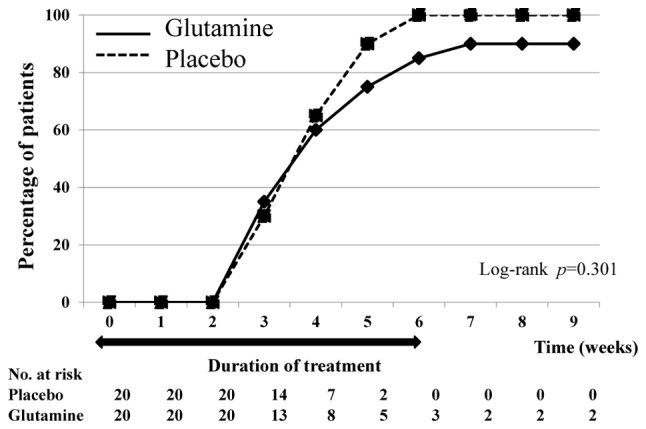
Cumulative incidence rate of severe oral mucositis. Kaplan-Meier method was used to analyze the time to onset of severe mucositis (≥G3, NCI CTCAE ver. 3.0) and the curves were compared using stratified log-rank tests. NCI CTCAE ver. 3.0, National Cancer Institute Common Terminology Criteria for Adverse Events version 3.0.

**Figure 3:**
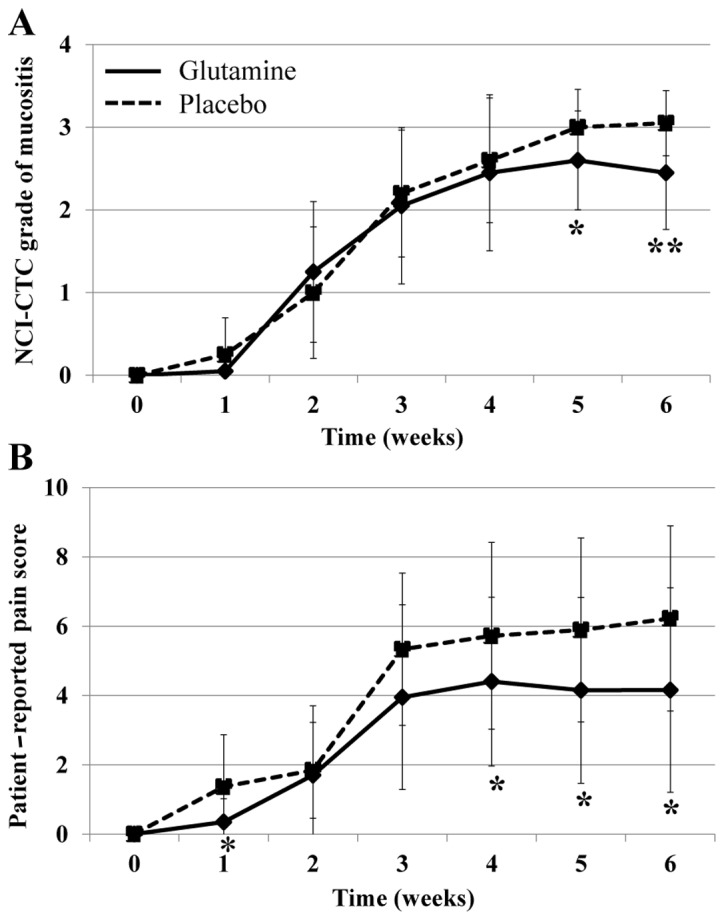
(A) Mean NCI CTCAE grade of mucositis. The NCI CTCAE grades were determined by two well-trained physicians weekly throughout CRT. (B) Mean patient-reported pain score. NRS was used as a patient-reported pain score. Patients assessed the strongest pain experienced during a day on a 0–10 scale. Both NCI CTCAE grades and NRS scores were analyzed using Mann-Whitney U test. ^*^p<0.05; ^**^p<0.01 vs. group P. NCI CTCAE, National Cancer Institute Common Terminology Criteria for Adverse Events; NRS, numerical rating scale.

Mucositis developed in all patients in both groups. A maximal mucositis grade of G4 was observed in 0 and 25% group G and P patients, respectively, while that of G2 was observed in 10 and 0% group G and P patients, respectively. Glutamine significantly decreased the mean maximal grade (group G, 2.9±0.3; group P, 3.3±0.4; p=0.005; Table II). The mean time to mucositis onset was 2.3±0.8 and 2.1±0.8 weeks (p=0.663), while the mean mucositis duration was 4.8±0.9 and 5.0±0.8 weeks in groups G and P, respectively (p=0.617). The mean time to severe mucositis onset (≥G3) was 4.2±1.1 and 4.2±1.0 weeks (p=0.829), while the mean severe mucositis (≥G3) duration was 2.2±1.4 and 2.8±1.1 weeks in groups G and P, respectively (Fig. 2). The mean mucositis grade as assessed by experienced physicians was significantly lower in group G than in group P at weeks 5 and 6 (p=0.027, p=0.002, respectively; Fig. 3A).

### NRS scores and rate of opioid usage

NRS scores were significantly lower in group G than in group P at weeks 4, 5 and 6 (p=0.049, p=0.019, p=0.032, respectively; Fig. 3B). In total, 17 (85%) and 19 patients (95%) used an opioid analgesic in addition to a non-steroidal anti-inflammatory drug (NSAID) in groups G and P, respectively. The mean duration of opioid use was significantly shorter in group G than in group P (group G, 19±11 days; group P, 28±14 days; p=0.029). The mean total opioid dose (in morphine equivalents) was 2,370±2,237 mg in group G and 3,959±3,566 mg in group P (p=0.101; Table III).

### Requirement and duration of nutritional support

Supplemental nutrition through a nasogastric tube, gastric fistula or intravenous peripheral parenteral nutrition was administered to 16 (80%) and 19 patients (95%) in groups G and P, respectively. The mean duration of supplemental nutrition required due to severe mucositis was significantly shorter in group G than in group P (group G, 18±13; group P, 27±11; p=0.046; Table III). The mean percentage change in body weight from baseline to week 8 was 3.6 and 6.0% in groups G and P, respectively. Changes in average BMI were similar between groups (21.6±3.2 at baseline and 20.2±2.9 at week 8 in group G and 22.1±3.9 at baseline and 20.7±3.2 at week 8 in group P). The mean daily intake of calories was 1,417±198 and 1,397±232 kcal in groups G and P, respectively (p=0.771; Table I).

### Biochemical blood analyses

The results of hematological and biochemical blood tests for renal function, liver function, lipids, C-reactive protein, rapid turnover protein and trace elements were comparable between groups (Table IV).

### Adverse effects of CRT

Severe mucositis interrupted CRT in 3 patients (15%) in group P but no patient in group G. There were no adverse effects and abnormal laboratory findings in group G. The amino acid profiles showed that glutamine did not affect the overall amino acid balance (data not shown).

### Influence of glutamine on the primary treatment outcome

After 10 weeks of treatment, tumor response was evaluated using PET-CT and histopathological examination of biopsy specimens. The tumor responses in group G were as follows: complete response (CR), 65%, 13/20; partial response (PR), 25%, 5/20; stable disease (SD), 10%, 2/20; and progressive disease (PD), 0%, 0/20. Those in group P were as follows: CR, 60%, 12/20; PR, 25%, 5/20; SD, 0%, 0/20; PD, 10%, 2/20; and unknown, 5%, 1/20. The response rate was 90% in group G and 85% in group P.

## Discussion

The present study demonstrated that glutamine significantly decreased the severity of CRT-induced mucositis in patients with HNC. Glutamine has important and unique metabolic properties. Free and abundant glutamine in the circulation as well as intracellular pools is essential for DNA synthesis, cell division and cell growth, all of which are necessary for wound healing and tissue repair (18). Glutamine participates in protein synthesis and extracellular matrix formation. Some studies have shown that glutamine increases collagen synthesis in human fibroblasts by a direct stimulatory effect and as a proline and hydroxyproline residue precursor (19,20). It also enhances the immune system and is an important fuel for both macrophages and lymphocytes. Intravenous glutamine supplementation reportedly increased IgA production in rats (21). Furthermore, glutamine has antioxidant properties as a glutathione precursor. Leitao *et al* showed that glutamine or alanyl glutamine accelerated mucosal remodeling from 5-fluorouracil-induced OM by increasing glutathione stores in hamster mucosa (22). Nose *et al* demonstrated that bolus enteral glutamine prevented cisplatin-induced intestinal mucosal injury in rats, possibly resulting in increased intracellular glutathione (23). Several clinical studies have shown the protective effects of glutamine on the mucosal epithelium (24–26). Topkan *et al* reported that oral glutamine decreased the incidence and duration of acute radiation-induced esophagitis in non-small cell lung cancer patients treated with radiotherapy (24). Cerchietti *et al* demonstrated that intravenous L-alanyl-L-glutamine significantly decreased the severity of CRT-induced OM in patients with HNC (25). Intravenous L-alanyl-L-glutamine is effective, yet in terms of availability and cost, there are some limitations to its widespread use for OM treatment. Recombinant human keratinocyte growth factor has also been shown to be effective in reducing severe OM (27), but it has been reported to cause several adverse effects and its high cost renders it inaccessible to most patients. In the present study, we hypothesized that oral glutamine decreased mucositis severity due to its multiple properties that affect the mucositis healing process.

A major finding of the present study was that glutamine considerably decreased the incidence of grade 4 mucositis, which is the most aggressive form of mucositis often resulting in unavoidable therapy interruption. Treatment delay caused by mucositis was observed in 0 patients in group G and in 15% patients in group P. During the 6-week CRT regimen, weeks 5 and 6 were the most debilitating, during which patients continuously consumed opioid analgesics with NSAIDs and received parenteral or enteral nutrition through a nasogastric tube or gastric fistula due to feeding difficulties caused by severe pain. Glutamine significantly decreased the mean maximal mucositis grade at weeks 5 and 6 as well as NRS scores at weeks 4, 5 and 6. Furthermore, physician-assessed mucositis correlated with NRS score and the duration of supplemental nutrition and opioid use was significantly lower in group G than in group P.

Several studies tested whether glutamine decreased the incidence and severity of CRT-induced OM in bone marrow transplant (BMT) and hematopoietic cell transplant (HCT) populations (14,28–34); some of these clinical trials, however, did not show a convincing benefit of glutamine from the perspective of decreasing OM incidence and severity (14,34). These studies could not perform conclusive evaluations of the effects of glutamine in the prevention and treatment of OM due to the small sample size, inadequate design and suboptimal glutamine doses (14,15,35). Further study is required to determine the effectiveness of oral glutamine supplementation in BMT and HCT patients. In terms of severity and location of mucositis, the differences between OM induced by local irradiation in HNC patients and that induced by systemic irradiation in BMT or HCT patients should be considered. During radiotherapy for HNC, a large quantity of radiation is more likely to act directly on tumor cells in the head and neck area, and although the damage is localized, it is more severe in these patients.

The aim of this double-blind, randomized, placebo-controlled trial was to determine whether oral glutamine decreased the severity of mucositis in HNC patients. To our knowledge, this is the first well-designed randomized controlled trial in this field. Untreated newly diagnosed HNC patients were enrolled and treated with identical CRT regimens. Physicians well-versed in mucositis improved reliability of the data. Nurses were responsible for distributing each dose to patients, and a pharmacist managed patient compliance, which was 99.6%. Poor nutritional status is expected to interfere with mucosal healing and regeneration by decreasing infiltration by immunocompetent cells (36). An independent NST, which was indispensable in the present study, evaluated patient nutritional status and the requirement for enteral feeding in both groups throughout treatment. The enteral nutrition administered in this study did not contain glutamine.

There were no significant differences between groups in weight loss, BMI and biochemical parameters such as albumin, transthyretin, transferrin, retinol-binding protein, iron, copper, zinc and amino acids throughout this study. Nutritional management by the NST maximized the non-nutritional effects of glutamine and enhanced the quality of the present study. In addition, there are several previous studies on mucositis, but each study evaluated mucositis induced by a variety of chemotherapeutic agents and the regimen was not unified. The incidence and severity of mucositis must be altered by chemotherapeutic agents which were actually used. In the present study, all patients were treated the completely same CRT regimen, and when the regimen had been changed, the patients were excluded in the statistical analysis. Our results should be considered reliable since all patients in both groups had the same opportunity to develop clinically significant mucositis; consequently, any differences in treatment outcome must be the result of glutamine supplementation.

There were no adverse events associated with glutamine intake, such as constipation, dry mouth or nausea. The clinical data did not show any differences in serum ammonia levels and amino acid profiles between groups. Notably, glutamine did not interfere with the therapeutic effects of CRT. The present study demonstrated that glutamine can be used safely in combination with CRT.

Mucositis, also known as mucosal barrier injury, is a biologically complex process involving several phases (37). Initially, mucosal injury is induced by oxidative stress and reactive oxygen species (ROS) generated by radiation and cisplatin. Glutamine is a precursor of glutathione, a major intracellular antioxidant protecting cells against oxidative stress. Exogenous glutamine administration restores tissue glutathione levels that are depleted after chemotherapy (22). We hypothesized that glutamine protected the mucosal epithelium from oxidative stress without a negative impact on tumor regression. Next, the ulcerative phase is associated most consistently with mucositis. This phase is symptomatic and carries a risk of bacterial translocation. There were no patients with grade 4 mucositis who presented with symptomatic bleeding from ulcers in this study. Glutamine reportedly suppresses ulcer formation (38). Finally, in the regeneration phase, it is necessary to have ample nitrogen supply for DNA synthesis and collagen for extracellular matrix formation. As a collagen precursor, hydroxyproline, which is indispensable for wound healing, is synthesized from glutamate, which is derived from glutamine.

In conclusion, the present study demonstrated that glutamine significantly decreases the severity of CRT-induced mucositis in HNC cancer patients. An integrative and multidisciplinary approach can result in substantial advances in the outcome of cancer therapy and improvement in patient quality of life.

## Figures and Tables

**Table I:** Patient characteristics.

	Glutamine group (n=20)	Placebo group (n=20)	P-value
Male/female	17/3	17/3	1
Age (years)	60.5±10.8	63.2±5.4	0.3330
Primary tumor location			
Nasopharynx	0 (0%)	2 (10%)	0.3755
Oropharynx	10 (50%)	6 (30%)	
Hypopharynx	7 (35%)	7 (35%)	
Larynx	3 (15%)	5 (25%)	
Stage			1
I	0 (0%)	0 (0%)	
II	2 (10%)	3 (15%)	
III	4 (20%)	3 (15%)	
IV	14 (70%)	14 (70%)	
ECOG performance status			
0	17 (85%)	19 (95%)	0.6050
1	3 (15%)	1 (5%)	
Total dose of radiation			1
66 Gy	18 (90%)	19 (95%)	
70 Gy	2 (10%)	1 (5%)	
Total dose of chemotherapeutic agents (mg)			
Cisplatin	193.3±19.2	187.2±21.3	0.3573
Docetaxel	97.7±8.8	93.9±10.5	0.2297
Diabetes mellitus	1 (5%)	3 (15%)	0.6050
Body mass index (kg/m^2^)	21.6±3.2	22.1±3.9	0.4282
Daily intake of calories (kcal/day)	1,417±198	1,397±232	0.7709

Gy, grays; ECOG, Eastern Cooperative Oncology Group. Values of age, total dose of chemotherapeutic agents, body mass index are means ± SD and analyzed by Welch’s t-test. Values of gender, primary tumor location, stage, total dose of radiation, diabetes are patient number and percentage and were analyzed by Fisher’s exact test.

**Table II:** Incidence and severity of mucositis.

	Glutamine (n=20)	Placebo (n=20)	P-value	Effect size
Incidence of mucositis	20 (100%)	20 (100%)	1	
Maximum grade of mucositis			0.0234^a^	
None	0	0		
Grade 1	0	0		
Grade 2	2	0		
Grade 3	18	15		
Grade 4	0	5		
Average grade of mucositis	2.9±0.3	3.3±0.4	0.0049^b^	0.89

Continuous variables were analyzed by Mann-Whitney U test and categorical variables were analyzed by Fisher’s exact test.

a p<0.05,

b p<0.01 vs. group P.

**Table III:** Incidence of opioid use and supplemental nutrition due to mucositis.

	Glutamine (n=20)	Placebo (n=20)	P-value	Effect size
Incidence of opioid use	17 (85%)	19 (95%)	0.6050	-
Time to onset of opioid use (days)	24±9	19±9	0.1011	0.56
Duration of opioid use (days)	19±11	28±14	0.0294^a^	0.69
Total dose of opioids (mg), morphine equivalents	2,370±2,237	3,959±3,566	0.1012	0.52
Incidence of supplemental nutrition	16 (80%)	19 (95%)	0.3416	-
Time to onset of administration of supplemental nutrition (days)	24±11	21±8	0.3867	0.35
Duration of supplemental nutrition (days)	18±13	27±11	0.0455^a^	0.69

Continuous variables were analyzed by Welch’s t-test and categorical variables were analyzed by Fisher’s exact test.

a p<0.05 vs. group P.

**Table IV:** Biochemical data of glutamine and placebo group.

	Baseline		After treatment	
		
	Glutamine group (n=20)	Placebo group (n=20)	Glutamine group (n=20)	Placebo group (n=20)
White blood cell count (×10^3^/mm^3^)	6.2±1.5	5.8±1.3	3.8±1.6	4.2±2.0
Total neutrophil count (×10^3^/mm^3^)	4,075±1,170	3,689±1,211	2,805±1,459	3,157±2,095
Total lymphocyte count (×10^3^/mm^3^)	1,360±481	1,430±312	424±171	520±204
Red blood cell count (×10^6^/mm^3^)	4.2±0.5	4.1±0.5	3.4±0.5	3.3±0.3
Hemoglobin, g/dl	13.2±1.7	13.1±1.7	10.1±0.5	10.7±1.0
Platelet count (×10^3^/mm^3^)	220.3±77.3	248.4±83.3	250.1±63.4	261.3±105.0
Blood urea nitrogen, mg/dl	14.0±4.8	13.8±3.1	19.0±9.8	18.7±6.3
Creatinine, mg/dl	0.7±0.2	0.7±0.2	0.8±0.2	0.8±0.2
Asparatate aminotransferase, U/l	24±18	26±16	17±5	19±5
Alanine aminotransferase, U/l	25±28	19±9	16±5	16±5
γ-glutamyl transpeptidase, U/l	64±87	44±36	48±40	63±63
Cholinesterase, U/l	282±90	273±74	256±83	244±91
Creatine phosphokinase, U/l	59±17	64±21	72±58	52±31
Total cholesterol, mg/dl	182±28	170±45	159±23	153±33
Total bilirubin, mg/dl	0.7±0.3	0.6±0.1	0.3±0.1	0.4±0.1
C-reactive protein, mg/dl	0.5±0.6	0.5±0.6	2.3±2.2	2.8±3.1
Albumin, g/dl	3.8±0.4	3.7±0.4	3.3±0.4	3.1±0.6
Transferrin, mg/dl	222±26^a^	194±28	203±25	172±26
Transthyretin, mg/dl	26±8	22±7	19±4	17±8
Retinol-binding protein, mg/dl	4.1±1.3^a^	3.2±1.0	3.4±0.9	3.1±1.4
Iron, μg/dl	85±41	77±31	67±29	69±21
Copper, μg/dl	119±25	124±25	143±46	149±24
Zinc, μg/dl	81±18	75±8	76±14	82±17

Values are means ± SD.

a p<0.05 vs. baseline of placebo group analyzed by Welch’s t-test.
